# Are Lumen-Apposing Metal Stents More Effective Than Plastic Stents for the Management of Pancreatic Fluid Collections: An Updated Systematic Review and Meta-analysis

**DOI:** 10.1155/2020/4952721

**Published:** 2020-04-20

**Authors:** Shali Tan, Chunyu Zhong, Yutang Ren, Xujuan Luo, Jin Xu, Yan Peng, Xiangsheng Fu, Xiaowei Tang

**Affiliations:** ^1^Department of Gastroenterology, Affiliated Hospital of Southwest Medical University, Luzhou, China; ^2^Department of Gastroenterology, The People's Hospital of Lianshui, Huaian, China; ^3^Department of Gastroenterology, Affiliated Hospital of North Sichuan Medical College, Nanchong, China

## Abstract

**Background and Aims:**

Recently, a new type of metal stent, named lumen-apposing metal stents (LAMS), has been designed to manage pancreatic fluid collections (PFC), and a few studies have reported its efficacy and safety. Therefore, we conducted this meta-analysis to investigate the role of LAMS for PFC.

**Methods:**

We searched the studies from PubMed, MEDLINE, Embase, and Cochrane databases from inception to May 2019. We extracted the data and analyzed the technical success, clinical success, and adverse events of LAMS to evaluate its efficacy and safety.

**Results:**

Twenty studies with 1534 patients were included. The pooled technical success, clinical success, and adverse event rates of LAMS for PFC were 96.2% (95% confidence interval (CI): 94.6%-97.4%), 86.8% (95% CI: 83.1%-89.8%), and 20.7% (95% CI: 16.1%-26.1%), respectively. Eight studies including 875 patients compared the clinical outcomes of LAMS with plastic stents. The pooled risk ratio (RR) of technical success and clinical success for LAMS and plastic stent was 1.01 (95% CI: 0.98-1.04, *P* = 0.62) and 1.06 (95% CI: 1.01-1.12, *P* = 0.03), respectively. As for the overall adverse events, the pooled RR was 1.51 (95% CI: 0.67-3.44, *P* = 0.32).

**Conclusions:**

Our current study revealed that LAMS has advantages over plastic stents for PFC, with higher clinical success rate and lower complication rate of infection and occlusion.

## 1. Introduction

Pancreatic fluid collection (PFC) is a common complication of pancreatitis. According to revised Atlanta Criteria, PFC can be divided into pancreatic pseudocysts (PPs) and walled-off necrosis (WON) [1]. Traditionally, PFC has been treated by surgical and percutaneous drainage. However, due to the limitations associated with these techniques [2–4] and recent advancements in minimally invasive techniques, endoscopic ultrasound- (EUS-) guided transmural drainage has emerged as a new form of therapy for PFC [5, 6]. Compared with surgical drainage, EUS offers a more precise visualization of the surrounding vessels, organs, and fluid collections. In addition, EUS can reduce the rate of adverse events [7]. In a previous study, Khan et al. reported that EUS-guided transmural drainage conveys several advantages, including a significantly higher rate of clinical success, reduced rates of reintervention, and a shorter period of hospitalization in comparison with percutaneous drainage [8].

Over the last decade, EUS-guided drainage has been conventionally performed for PFC with a plastic stent and a fully covered self-expanding metal stent. However, more recently, a dedicated device, a lumen-apposing metal stent (LAMS), has been developed as an alternative for PFC. Owing to its larger diameter and its biflanged wide lumen, the LAMS is less likely to cause occlusion, thus reducing the need for repetitive stent alterations. A number of studies have since shown that LAMS provides an excellent tool for PFC drainage and has several clinical advantages over plastic stents [9, 10]. Most recently, a meta-analysis conducted by Hammad et al. in 2017 [11] demonstrated that LAMS has better efficacy and safety over plastic stents for PFC. However, other studies have revealed that the efficiency of LAMS is not significantly different to that of conventional stents [12, 13]. Furthermore, LAMS has a high risk of complications [14]. Therefore, we performed this updated meta-analysis to evaluate the precise role of LAMS for PFC.

## 2. Methods and Materials

### 2.1. Study Design

In May 2019, we conducted a meta-analysis, in accordance with the checklist of the Preferred Reporting Items for Systematic Reviews and Meta-Analyses (PRISMA), to summarize the available data relating to the management of PFC with LAMS [15]. Two independent reviewers screened the retrieved citations, selected the eligible studies, and extracted the data for analysis. Any discrepancy was discussed.

### 2.2. Search Strategy

A literature review was conducted in PubMed, MEDLINE, Embase, and Cochrane databases, to identify studies related to the endoscopic management of PFC. The search terms for PubMed were focused on lumen-apposing metal stents, pancreatic fluid collections, metal stents, pancreatic pseudocyst, walled-off necrosis, AXIOS, LAMS, and WON (Table S1). These terms were adapted for use with other databases. We also screened the reference lists of all included to identify additional studies of relevance. For each article, two independent reviewers evaluated the title, abstract, and full text.

### 2.3. Eligibility

The full text of all selected studies was screened in strict accordance with specific inclusion and exclusion criteria, which were predefined by two investigators. The inclusion criteria for this meta-analysis were as follows: (1) retrospective, prospective, case-control, or cohort studies and clinical trials (including randomized controlled trials) and (2) studies reporting the clinical outcomes of LAMS in the treatment of PFC. The exclusion criteria were (1) animal studies; (2) case reports; (3) fewer than 10 patients included; (4) commentaries, reviews, conference abstracts, or surveys; and (5) publications in a language other than English. For overlapping publications from the same center, only the most recent and comprehensive publication was considered for inclusion.

### 2.4. Quality of Studies

Methodological quality was evaluated by two investigators. The risk of bias for individual studies were assessed by the Newcastle-Ottawa Quality Assessment Scale for nonrandomized studies [16, 17] and the Jadad scale for randomized controlled trials [18] (Table 1). All of the studies included in the meta-analysis were categorized into high quality, medium quality, and low quality. Discrepancies were resolved by a discussion between the two investigators.

### 2.5. Endpoint Definition and Statistical Analysis

Two investigators separately extracted a range of data, including the baseline characteristics of the included studies (author name, country, year of publication, type of study, sample size, age and gender), clinical characteristics of PFC (etiology, type of PFC, size and location of the PFC, intervention, and follow-up), and a summary of the study results (technical success, clinical success, adverse event, and DEN (direct endoscopic necrosectomy) rates). Plastic stents and LAMS were analyzed with respect to the primary endpoints of technical and clinical success. The definition of clinical success was “resolution of clinical symptoms and a reduced PFC size on imaging.” The definition of technical success was “successful placement of the stent”. The secondary outcomes were the rates of adverse events. Dichotomous data were analyzed by using the risk ratio (RR) and the pooled event rate with 95% confidence intervals (CI). To examine the heterogeneity of the included studies, we used Cochran's Q statistic and the *I*^2^ test. When the *P* value was < 0.05 (Q statistic) and/or *I*^2^> 50%, we adopted the random effects model on account of significant levels of heterogeneity. Otherwise, we selected the fixed effects model. We also carried out a subgroup analysis according the different types of PFC. The possibility of publication bias was assessed via funnel plots and then confirmed statistically by Egger's regression test. Sensitivity analyses were also performed by systematically removing each study in turn to explore its effect. All statistical analyses were performed by Review Manager 5.3 (RevMan; The Cochrane Collaboration, Oxford, United Kingdom) and Comprehensive Meta-Analysis, version 2 (Biostat, Englewood, NJ, USA).

## 3. Results

### 3.1. Study Characteristics

**Figure 1:**
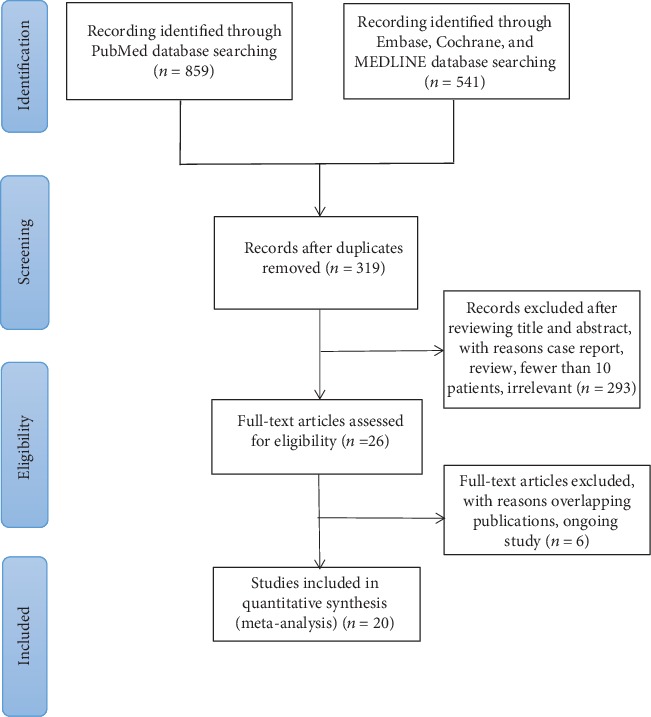
PSISMA flowchart.

Our literature searches led to the identification of 1400 articles. After screening the titles and abstracts, 20 studies were found to meet our eligibility criteria (Figure 1) [3, 9, 10, 12–14, 19–32]. A total of 1534 patients were included. All of the studies included in our meta-analysis were published between 2015 and 2019.  Table 2 summarizes the characteristics of a single-arm study featuring 659 patients that qualified for this meta-analysis. The causes for PFC were mainly gallstones (37.8%), alcohol (26.4%), idiopathy (13.6%), and others (22.2%). Mean follow-up time ranged from 84 days to 426.5 days. All of the single-arm studies used the AXIOS lumen-apposing metal stent for drainage except one.  Table 3 shows the clinical results of this single-arm study, including the rates of technical success, clinical success, adverse events, and DEN.

Further details of studies comparing LAMS with the plastic stent that were included in the study are presented in Table 4. Of the eight studies, the numbers of patients in the plastic and LAMS arm groups were 530 and 345, respectively. In the LAMS group, the age of patients ranged from 45.4 to 55.8 years. PFC dimensions varied from 8.01 to 12.0 cm. In the group of patients treated with plastic stents, age varied from 46.6 to 60.3 years. Lesion dimensions ranged from 6.98 to 10.9 cm. There were no significant differences with regard to the fundamental characteristics of the two stent groups in most of the included studies.

Quality assessments are reported in Table 1. One study scored 5 on the quality score and was deemed to be of low quality. Nine studies had a score of 6 or 7 and were regarded to be of medium quality. The other nine studies achieved a high score (>7) and therefore showed satisfactory high quality. The quality assessment of one randomized trial was performed using the Jadad scale [12]; this trial had a Jadad score of 3 and was therefore considered to be of high quality.

### 3.2. Technical Success of LAMS

**Figure 2:**
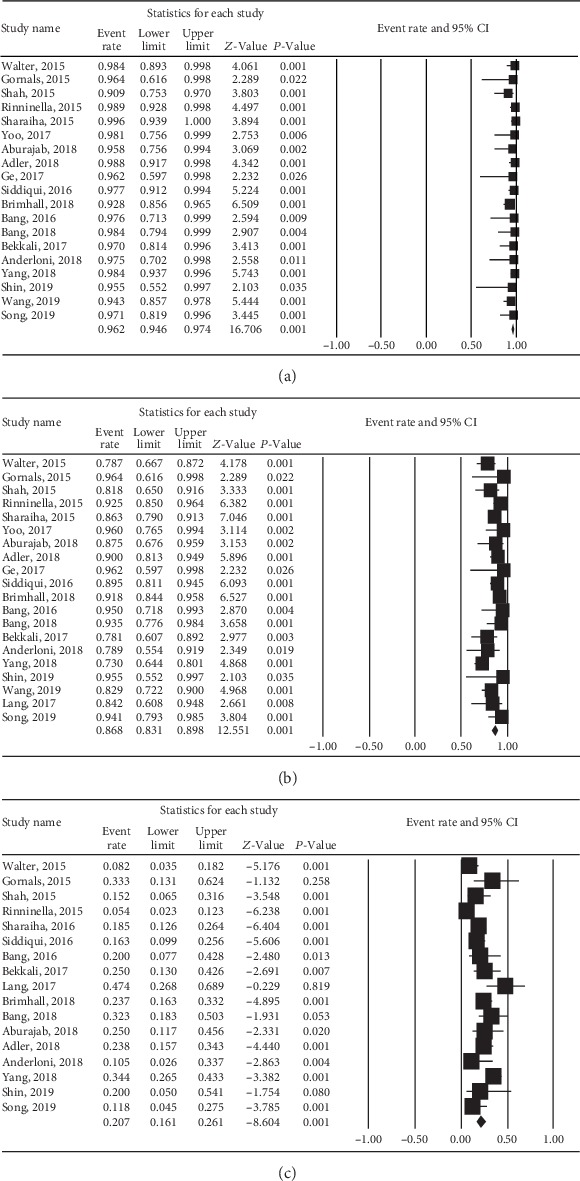
Forest plot of technical success, clinical success, and adverse events for lumen-apposing metal stents (LAMS) in management of pancreatic fluid collections (PFC). (a) Technical success. (b) Clinical success. (c) Adverse events.

Nineteen studies investigated the technical success of PFC drainage with LAMS; success rates ranged from 91% to 100%. As shown in Figure 2(a), the pooled event rate for the technical success of LAMS was 96.2% (95% CI: 94.6%-97.4%). These results did not change after removing the largest study to test whether it exerted influence over the general findings. A low degree of heterogeneity (*Q* = 15.26, *P* = 0.64, *I*^2^ = 0%) was evident across these 19 studies.

### 3.3. Clinical Success of LAMS

Twenty studies reported the clinical success rate of LAMS for PFC; these rates ranged from 73% to 100%. As shown in Figure 2(b), the pooled clinical success rate was 86.8% (95% CI: 83.1%-89.8%). Removing the largest study did not change the overall findings. There was a moderate degree of heterogeneity (*Q* = 37.87, *P* = 0.006, *I*^2^ = 49.8%) among these 20 studies.

### 3.4. Adverse Events

The rate of adverse events when using LAMS for PFC across all of the studies shown in Figure 2(c). The pooled event rate for adverse events associated with LAMS was 20.7% (95% CI: 16.1-26.1%). Removing the largest study did not change the overall findings. A high degree of heterogeneity (*Q* = 45.19, *P* < 0.001, *I*^2^ = 64.6%) was evident among the included studies. The detailed adverse events for the use of LAMS to treat PFC are shown in Tables S2, 3.

### 3.5. Sensitivity Analyses

Removing one study at a time from the analysis did not significantly affect the overall effect size or the heterogeneity for any of the outcomes. The largest change occurred when we removed the data reported by Yang et al. with regard to primary outcome (clinical success rate); this reduced the level of heterogeneity from moderate to low and changed the overall effect size from 86.8% to 87.4% [30]. These results suggested that no single study could significantly influence the pooled outcomes.

### 3.6. Meta-analysis

**Figure 3:**
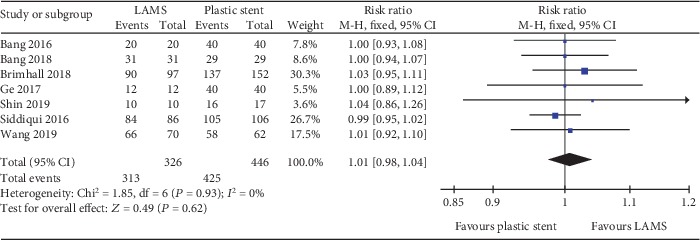
Forest plot to compare technical success between lumen-apposing metal stents (LAMS) and plastic stents for drainage of pancreatic fluid collections (PFC).

**Figure 4:**
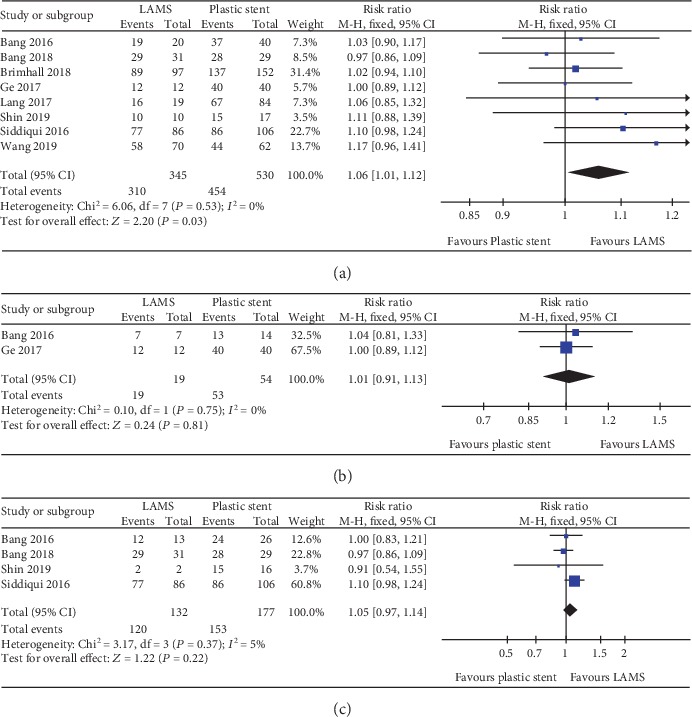
Forest plot to compare clinical success between lumen-apposing metal stents (LAMS) and plastic stents for drainage of pancreatic fluid collections (PFC). (a) Overall clinical success. (b) Pancreatic pseudocyst (PP). (c) Walled-off necrosis (WON).

**Figure 5:**
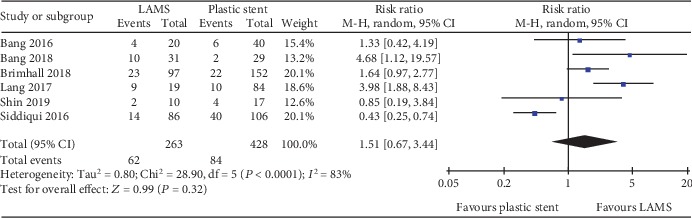
Forest plot to compare overall adverse events between lumen-apposing metal stents (LAMS) and plastic stents for drainage of pancreatic fluid collections (PFC).

**Figure 6:**
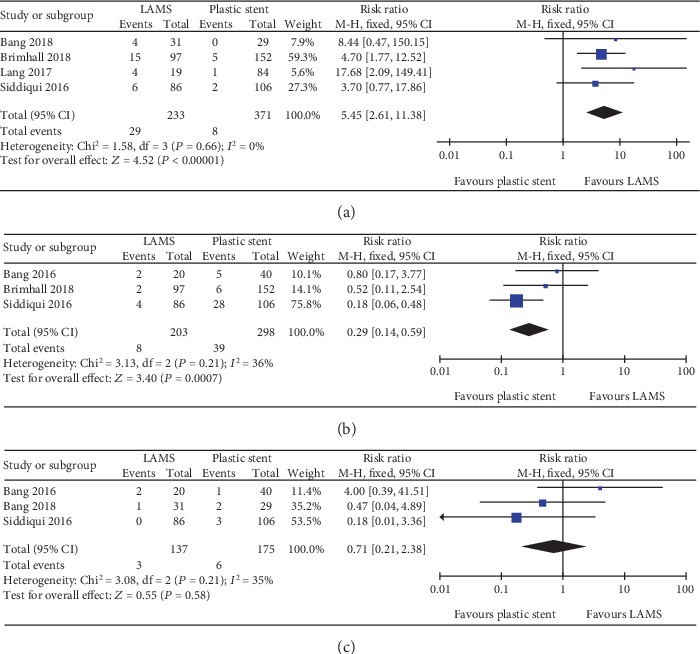
Forest plot to compare common adverse events between lumen-apposing metal stents (LAMS) and plastic stents for drainage of pancreatic fluid collections. (a) Bleeding. (b) Postprocedural infection/occlusion. (c) Migration.

A total of seven studies with 772 patients and eight studies with 875 patients were compared for LAMS and plastic stent with regard to technical success and clinical success, respectively. The pooled RR for technical success was 1.01 (95% CI: 0.98–1.04; *P* = 0.62; *I*^2^ = 0%) (Figure 3). For clinical success, the pooled RR was 1.06 (95% CI: 1.01–1.12; *P* = 0.03; *I*^2^ = 0%) (Figure 4(a)). Subgroup analyses were performed to compare the clinical success rates of patients with PP or WON. In the subgroup analysis involving PP (two studies, 73 patients), the clinical success rate of the LAMS group (19/19; 100%) was comparable to the plastic stent group (53/54; 98%) (RR 1.01; 95% CI: 0.91–1.13), and no heterogeneity was evident between the studies (*I*^2^ = 0%; *P* = 0.75) (Figure 4(b)). In the subgroup analysis involving WON (four studies, 309 patients), there was no significant difference between the LAMS group (120/132; 90.9%) and the plastic stent group (153/177; 86.4%); the pooled RR was 1.05 (95% CI: 0.97–1.14), and low levels of heterogeneity were found among the studies (*I*^2^ = 5%; *P* = 0.37) (Figure 4(c)). We also compared the overall adverse events of these two stents; the pool RR was 1.51 (95% CI: 0.67–3.44; *P* = 0.32; *I*^2^ = 83%) (Figure 5). In addition, we conducted a subgroup analysis for major complication events, including bleeding, postprocedural infection, and occlusion and migration between the two groups. The pooled RR for bleeding was 5.45 (95% CI: 2.61–11.38; *P* < 0.001; *I*^2^ = 0%). For postprocedural infection and occlusion, the pooled RR was 0.29 (95% CI: 0.14–0.59; *P* = 0.0007; *I*^2^ = 36%). The pooled RR of migration was 0.71 (95% CI: 0.21–2.38; *P* = 0.58; *I*^2^ = 35%)(Figures 6(a)–6(c)).

### 3.7. Publication Bias

**Figure 7:**
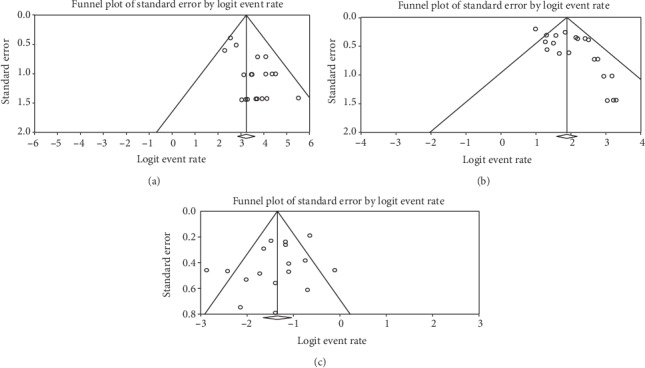
Funnel plot for publication bias of technical success, clinical success, and adverse events. (a) Technical success. (b) Clinical success. (c) Adverse event.

We investigated the risk of bias for technical success, clinical success, and adverse events. Considerable publication bias was observed for technical success (*P* = 0.002) and clinical success (*P* = 0.003). No publication bias was evident for adverse events (*P* = 0.16) (Figures 7(a)–7(c)).

## 4. Discussion

Over recent years, EUS-guided drainage for PFC has emerged as a less invasive alternative to surgery. The technique and devices used for the endoscopic drainage of PFC are constantly evolving. With the development of LAMS, the procedure for EUS-guided PFC drainage has been simplified and made more effective. The unique dog-bone design of the LAMS provides a stable anastomosis for the direct apposition of the two separate lumens. A fully covered stent maintains a stable conduit, thus reducing the risk of enteric contents leaking. Furthermore, the large diameter of the LAMS allows for more aggressive DEN and nasocystic drainage when used for PFC [33]. In the present study, our results showed that LAMS was associated with a high technical success rate (96.2%) with no heterogeneity, a high clinical success rate (86.8%) with moderate heterogeneity, and a low incidence of adverse events (20.7%) with high heterogeneity, when used to manage PFC. It appears that the wider diameter of LAMS might be a benefit for PFC, and thus results in improved clinical outcomes. Furthermore, we compared the efficacy and safety of LAMS and plastic stents. Our findings demonstrate the significant superiority of LAMS in comparison to plastic stents for PFC, with a significant higher clinical success rate (*P* = 0.03). Furthermore, a subgroup analysis showed that the clinical outcome of LAMS in WON was slightly better than the plastic stent, although this was not statistically significant (*P* = 0.22). These findings contradicted existing literature, which reports high efficacy (90%) for LAMS but lower efficacy (50-65%) for plastic stents in WON [34, 35]. In view of the small numbers of studies included in our study, our attempts to investigate these conditions separately via subgroup analysis should be interpreted with caution. Furthermore, it was difficult to draw specific conclusions for the subgroup analyses, although our results were in line with the only randomized controlled study published so far. In this particular study, Bang et al. demonstrated that there were no significant differences in clinical outcomes when compared between LAMS and plastic stents in the management of WON, except for procedure time [12].

With regard to complications, our data demonstrated that the most common adverse events associated with the use of LAMS for PFC were infection (7.2%), bleeding (5.1%), and migration (2.5%). Bleeding was reported in 29 of the 233 patients in the LAMS group and in 8 of the 371 patients in the plastic stent group. With regard to the pooled RR for bleeding rates between the two groups, we found that LAMS had a significantly higher risk than the plastic stent (*P* < 0.001); these findings were consistent with a previous study [36]. The underlying reason for this may be due to the fact that LAMS would hold their location by friction against regional blood vessels surrounding the necrotic cavity contributing to bleeding. In contrast, plastic stents tend to gravitate towards the gastrointestinal lumen after PFC has been resolved. In addition, the larger luminal area enables more gastric acid to enter into the PFC cavity; this may damage the blood vessels and promote bleeding. With regard to postprocedural infection and occlusion rates, our study showed that LAMS was superior to plastic stents in terms of infection events (*P* = 0.0007). Because of the wider lumen, LAMS can provide better access to the PFC cavity, thus facilitating further endoscopic intervention (direct necrosectomy), and reduces the risk of occlusion and infection; this cannot be accomplished with plastic stents, which have a small lumen. LAMS has been introduced for the management of PFC by virtue of its large diameter and biflared flanges, which may also reduce the rate of migration. However, our study failed to demonstrate that LAMS is associated with better outcomes than the plastic stent. The migration of LAMS was reported in three patients from the LAMS group, compared to six patients from the plastic stent group; this difference was not statistically significant (*P* = 0.58). However, this result was limited by the very small numbers of studies and sample sizes. In terms of overall adverse events, no significant differences were found between the two groups, although there was a high degree of heterogeneity. Although several studies reported that both early and delayed adverse events were associated with LAMS, we were not able to perform a subgroup analysis for adverse events on the basis of PFC subtype, due to the limited amount of data available.

To some extent, the results of our present meta-analysis were in line with a previous systematic review by Hammad et al. [11]; for example, we observed better clinical success and comparable technical success, for LAMS when compared to plastic stents. However, our study did not demonstrate the superiority of LAMS with regard to adverse events. Our experience and technical capability for the use of plastic stents and LAMS have improved significantly since 2017, and dedicated metal and plastic stents for PFC drainage have also become available. To further explore the reasons underlying such results in our study, we performed a subgroup analysis. LAMS is associated with a high risk of bleeding, and plastic stents are known to be prone to infection or occlusion, thus contributing to a comparable rate of adverse events. However, we felt that this previous review [11] was biased in favour of LAMS due to poor methodology. This previous review included studies that used biflanged metal stents, another form of metallic stent, and all of these studies involved only patients with WON [37–39]. Consequently, we thought that this particular study was not valid for comparison of LAMS and plastic stents for the drainage PFC.

There are several limitations to our meta-analysis that should be considered. First of all, the majority of these studies were retrospective, with only three prospective studies and one randomized controlled study. Consequently, we need to interpret our meta-analysis with caution. In addition, we cannot avoid the inherent methodological limitations of meta-analysis because of quality limitations and the quantity of the evidence available. Secondly, the definitions used for technical and clinical success differed across different studies. Most of these studies were retrospective, with small sample sizes. To partially eliminate this limitation, we excluded studies with fewer than 10 patients. Thirdly, there was a discrepancy with regard to the type of LAMS used; we therefore excluded studies relating to the Nagi stent. In addition, there was considerable heterogeneity among studies in the overall analysis with regard to clinical success rates and adverse events. Furthermore, DEN has been shown to contribute significantly to the clinical success of LAMS for the drainage of WON [10], although this work depended on the endoscopist and did not follow a specific protocol. Consequently, we did not pool this result in our meta-analysis. In addition, we detected a publication bias for both technical and clinical success. Publication bias can arise from language bias, inflated estimates by a flawed methodological design in smaller studies, or a lack of publication of small trials with opposite results and so on, which were unable to estimate. Finally, two recent studies reported that LAMS was more costly in the management of PFC [40, 41]. In our meta-analysis, we did not perform cost-effective analysis because such data were not commonly reported.

In conclusion, our current study revealed that LAMS had certain advantages over plastic stents in the management of PFC and was associated with higher clinical success rates and lower complication rates for infection and occlusion. Further randomized controlled trials, with large sample sizes and multiple centers, are now required to determine the precise role of LAMS and plastic stents and focus on identifying suitable subsets of patients for each technique.

## Figures and Tables

**Table 1:** Newcastle-Ottawa Quality Assessment Scale for cohort studies included in this review.

Study	Selection				Outcome assessment			Comparability		Quality of study
	1	2	3	4	1	2	3	1	2	
Bekkali et al. [19]	+	+	+	+	+	+	+	+	+	High quality
Walter et al. [20]	+	+	+	+	+	+	+			Medium quality
Wang et al. [21]	+	+	+	+	+	+		+	+	High quality
Gornals et al. [22]	+	+		+	+	+	+			Medium quality
Sharaiha et al. [23]	+	+	+	+	+	+		+	+	High quality
Ge et al. [9]	+			+	+	+	+			Low quality
Rinninella et al. [24].	+	+	+	+	+	+	+	+		High quality
Lang et al. [14]	+	+	+	+	+	+	+			Medium quality
Yoo et al. [25]	+	+	+	+	+	+	+	+	+	High quality
Siddiqui et al. [10]	+	+		+	+	+	+	+	+	High quality
Brimhall et al. [13]	+	+	+	+	+	+	+	+	+	High quality
Bang et al. [26]		+	+	+	+	+	+			Medium quality
Shah et al. [3]	+	+	+	+	+	+	+			Medium quality
Aburajab et al. [27]	+		+	+	+	+		+		Medium quality
Adler et al. [28]	+	+	+	+	+	+	+	+		High quality
Anderloni et al. [29]	+		+	+	+	+	+	+		Medium quality
Yang et al. [30]	+	+	+	+	+	+	+	+	+	High quality
Shin et al. [31]	+	+	+	+	+	+		+		Medium quality
Song et al. [32]	+		+	+	+	+	+			Medium quality

Selection: 1: representativeness of the exposed cohort; 2: selection of the nonexposed cohort; 3: ascertainment of exposure; 4: outcome of interest not present at start of study. Outcome assessment: 1: assessment of outcome; 2: adequacy of duration of follow-up; 3: adequacy of completeness of follow-up. Comparability: 1: study controls for confounder; 2: study controls for any additional factors.

**Table 2:** Characteristics of studies included in the meta-analysis (single arm).

Author, year, country	Design	No.	Age	Males	Etiologies	Type of PFC	Dimensions (cm)	Location of PFC	Intervention	Follow-up
Walter et al. [20], 2015, Netherlands	Prospective	61	55	38	Gallstones 19 Alcohol 22 Idiopathic 9 Postsurgical 6 Other 5	PP 15 WON 46	9 median	Head 7 Neck 4 Body 35 Tail 11 Entire 2	Hot-AXIOS 15 × 10 mm, 10 × 10 mm	NA
Gornals et al. [22], 2015, Spain	Retrospective	12	52.5	9	Alcohol 5 Idiopathic 2 Lithiasis 5	WON 13	12.4	NA	AXIOS 15 × 10 mm, 10 × 10 mm	13th month
Shah et al. [3], 2015, international	Prospective	33	53	18	Gallstones 6 Alcohol 6 Postsurgical 4 Idiopathic 15 Other 2	WON 11 PP 22	9	NA	AXIOS 15 × 10 mm, 10 × 10 mm	NA
Rinninella et al. [24], 2015, Italy	Retrospective	93	60	71	Gallstones 28 Alcohol 23 Idiopathic 17 Postsurgical 5 Chronic pancreatitis 13 Other 7	PP 18 WON 75	10 median	NA	Hot-AXIOS 15 × 10 mm, 10 × 10 mm	320th day
Sharaiha et al. [23], 2016, USA	Retrospective	124	54.2	75	Gallstones 59 Alcohol 25 Idiopathic 16 Trauma 6 Autoimmune 4 Other 14	WON 124	10.5	Head 14 Body/tail 110	AXIOS 15 × 10 mm, 10 × 10 mm	4th month
Yoo et al. [25], 2017, USA	Retrospective	25	50	14	Gallstones 10 Alcohol 7 Other 8	PP 3 WON 22	8.2	Head 3 Body/tail 22	AXIOS 15 × 10 mm, 10 × 10 mm	7.8th month
Bekkali et al. [19], 2017, UK	Retrospective	32	57	18	Gallstones 20 Alcohol 3 Other 9	WON 32	15	NA	Hot-AXIOS 15 × 10 mm	NA
Aburajab et al. [27], 2018, USA	Retrospective	24	54	17	Gallstones 8 Alcohol 9 Idiopathic 3 Other 4	PP 24	10	Head 2 Body/tail 22	AXIOS 15 × 10 mm	NA
Adler et al. [28], 2018, USA	Retrospective	80	53.1	48	Gallstone 39 Alcohol 24 Idiopathic 6 Drug 2 Autoimmune 1 Hypertriglyceridemia 8	PP 12 WON 68	11.8	Head 4 Body/tail 76	Cold-AXIOS, 15 × 10 mm, 10 × 10 mm	6th month
Anderloni et al. [29], 2018, Italy	Retrospective	19	64.3	7	Alcohol pancreatitis 2 Gallstone pancreatitis 10 Idiopathic pancreatitis 5 Postsurgical 2	PP 16 WON 3	10.2	NA	Hot-AXIOS 15 × 10 mm, 10 × 10 mm	426.5-day
Yang et al. [30], 2018, USA	Retrospective	122	50.9	79	NA	PP 58 WON 64	10.6	NA	Hot-AXIOS 15 × 10 mm, 10 × 10 mm	84th day
Song et al. [32],2019, Korea	Prospective	34	51.7	26	Gallstones 4 Alcohol 16 Postsurgical 13 Hypertriglyceridemia 1	PP 34	9.23	NA	Niti-S SPAXUS	NA

PFC: pancreatic fluid collections; PP: pancreatic pseudocyst; WON: walled-off necrosis.

**Table 3:** Summary of results from included studies (single arm).

Study	Technical success, *n* (%)	Clinical success, *n* (%)	Adverse events	DEN
Walter et al. [20], 2015, Netherlands	98% (60/61) (total)	WON 81% (35/43) PP 93% (13/14)	4 infection/occlusion 1 perforation	WON 15/35
Gornals et al. [22], 2015, Spain	WON 100% (13/13)	WON 100% (13/13)	2 bleeding 1 infection-stent migration 1 infection-stent occlusion	WON 13
Shah et al. [3], 2015, international	91% (30/33) (total)	81.8% (27/33) (total)	3 abdominal pain 1 stent migration and infection 1 stent dislodgement	11
Rinninella et al. [24], 2015, Italy	WON 98.7% (74/75) PP 100% (18/18)	WON 90.7% (68/75) PP 100% (18/18)	1 massive bleeding 1 perforation 1 pneumoperitoneum 1 postdrainage infection 1 stent displacement/migration	33
Sharaiha et al. [23], 2016, USA	WON 100% (124/124)	WON 86.3% (107/124)	7 stents migration 7 stent occlusion 7 infection 2 bleeding	78
Yoo et al. [25], 2017, USA	WON 100% (22/22) PP 100% (3/3)	WON 95.5% (21/22) PP 100% (3/3)	0	WON 1
Bekkali et al. [19], 2017, UK	WON 97% (32/33)	WON 78.1% (25/32)	1 stent misplacement 4 additional percutaneous drain 3 dislodged stent	NA
Aburajab et al. [27],, 2018, USA	PP 96% (23/24)	PP 91% (21/23)	1 perforation 4 infection 1 migration	NA
Adler DG et al. [28], 2018, USA	99% (79/80)	90% (72/80)	4 perforation 2 suprainfection 13 bleeding	63
Anderloni et al. [29], 2018, Italy	100% (19/19)	83.3% (15/19)	1 occlusion and infection 1 migration	NA
Yang et al. [30], 2018, USA	PP 98.3% (57/58) WON 98.4% (63/64)	PP 95.5% (55/58) WON 53.1% (34/64)	8 migration 28 occlusion 2 partially embedded stent 4 misdeployment	WON 23
Song et al. [32], 2019, Korea	PP 97.1% (33/34)	PP 94.1% (32/34)	1 maldeployment 3 infection	NA

DEN: direct endoscopic necrosectonomy; PP: pancreatic pseudocyst; WON: walled-off necrosis.

**Table 4:** Characteristics of studies included comparing LAMS versus plastic stent.

Author, year, country	Design	Groups	N	Age	Male	Type of PFC	Dimensions (cm)	Technical success	Clinical success	Adverse events	DEN
Ge et al. [9], 2017, China	Retrospective	LAMS Plastic	12 40	NA NA	NA NA	PP PP	NA NA	12 40	12 40	NA NA	NA NA
Lang et al. [14], 2017, USA	Retrospective	LAMS	19	54.6	10	PP 10	10.4	99% (total)	16	4 bleeding, 5 unplanned endoscopy	NA
						WON 9					
		Plastic	84	52.2	52	PP 70	8.8		67	8 unplanned endoscopy, 1 perforation, 1 bleeding	NA
						WON 14					
Siddiqui et al. [10], 2016, USA	Retrospective	LAMS Plastic	86 106	51.5 56.3	77 68	WON WON	11.4 10.6	84 105	77 86	6 bleeding, 1 suprainfection, 3 perforation, 3 stent occlusion, 1 abdominal pain 2 bleeding, 5 suprainfection, 1 perforation, 23 stent occlusion, 3 migration, 6 other	38 4
Brimhall et al. [13], 2018, USA	Retrospective	LAMS	97	47	65	PP 16	8.01	90	89	15 bleeding, 2 infection, 6 other	PP 2
						WON 81					WON 11
		Plastic	152	48	98	PP 36	6.98	137	137	5 bleeding, 5 perforation, 6 infection, 6 other	PP 2
						WON 116					WON 31
Bang et al. [26], 2016, USA	Retrospective	LAMS	20	50.7	11	PP 7	12.0	PP 7	PP 7	2 infection, 2 symptomatic migration	NA
						WON 13		WON 13	WON 12		
		Plastic	40	52.9	25	PP 14	10.9	PP 14	PP 13	5 infection, 1 symptomatic migration	NA
						WON 26		WON 26	WON 24		
Bang et al. [12], 2018, USA	RCT	LAMS Plastic	31 29	55.8 60.3	20 16	WON WON	10.2 10.7	31 29	29 28	4 bleeding, 2 buried stent, 3 stricture, 1 migration 2 migration	4 6
Shin et al. [31], 2019, Korea	Retrospective	LAMS	10	55.8	8	PP 8	8.28	PP 8	PP 8	1 abdominal pain, 1 pneumoperitoneum	NA
						WON 2		WON 2	WON 2		
		Plastic	17	56.4	14	WON 17	7.56	WON 16	WON 15	2 intraprocedural bleeding, 2 pneumoperitoneum	
Wang et al. [21], 2019, China	Retrospective	LAMS	70	45.4	52	PP 53	10.9	66	58	NA	NA
						WON 17					
		Plastic	62	46.6	36	PP 52	10.3	58	44	NA	
						WON 10					

LAMS: lumen-apposing metal stents; BFMS: biflanged metal stents; WON: walled-off necrosis; PP: pancreatic pseudocyst; DEN: direct endoscopic necrosectomy; RCT: randomized clinical trial.

## Supplementary Materials

Supplementary Materials Table S1: search strategy. Table S2: adverse event rate of lumen-apposing metal stents (LAMS). Table S3: major adverse events of the meta-analysis.
