# Supplementary material for: Are Lumen-Apposing Metal Stents More Effective Than Plastic Stents for the Management of Pancreatic Fluid Collections: An Updated Systematic Review and Meta-analysis
Source: Gastroenterol Res Pract. 2020 Apr 20;2020:4952721. doi: 10.1155/2020/4952721 (PMC7189322; doi:10.1155/2020/4952721)
Supplement: Supplementary Materials — Table S1: search strategy. Table S2: adverse event rate of lumen-apposing metal stents (LAMS). Table S3: major adverse events of the meta-analysis. [file 4952721.f1.pdf]

Table S1 Search strategy

Pubmed	pancreatic fluid collection OR peripancreatic-fluid-collection OR PFC OR Pancreatic-fluid-collection* OR peripancreatic-fluid-collection*<br>OR<br>walled-off-pancreatic-necros* OR WOPN OR walled-off-necros* OR WON OR necrotic-collection* OR cystic-collection*<br>OR<br>pancreatic pseudocyst [Mesh] OR Pancreatic Pseudocysts OR Pseudocyst, Pancreatic OR Pseudocysts, Pancreatic<br>AND<br>Lumen-apposing-metal-stent* OR lumen-apposing metallic-stent* OR lumen-apposing-stent* OR LAMS OR lumen-apposing-self-expandable-metallic-stent* OR lumen-apposing-self-expandable-metal-stent* OR AXIOS OR metal stent* OR Stents [Mesh] OR Stent
Embase	‘pancreas pseudocyst’/exp OR ‘pancreas abscess’/exp OR Pancreatic-fuid-collection* OR walled-of-pancreatic-necros* OR WOPN OR pancreatic-abscess* OR peripancreatic-fuid-collection*<br>OR<br>((Pancreas OR pancreatic OR intrapancreatic OR peripancreatic) AND (pseudocyst* OR walled-of-necros* OR WON OR necrotic-collection* OR cystic-collection*))

	AND<br>Lumen-apposing-metal-stent* OR lumen-apposing metallic-stent* OR lumen-apposing-stent* OR LAMS OR lumen-apposing-self-expandable-metallic-stent* OR lumen-apposing-self-expandable-metal-stent* OR LASEMS OR self-expandable-metal-stent* OR self-expandable-metallic-stent* OR self-expanding-metal stent* OR self-expanding-metallic-stent* OR AXIOS
Medline	Lumen-apposing-metal-stent* [tiab] OR lumen-apposing metallic-stent* [tiab] OR metal stent* [tiab] OR stent* [tiab] OR LAMS [tiab] OR AXIOS [tiab]<br>AND<br>pancreatic fluid collection [tiab] OR peripancreatic-fluid-collection [tiab] OR PFC OR Pancreatic-fluid-collection* [tiab] OR peripancreatic-fluid-collection* [tiab]
Cochrane	pancreatic fluid collection OR peripancreatic-fluid-collection OR PFC OR Pancreatic-fluid-collection* OR peripancreatic-fluid-collection*<br>OR<br>walled-off-pancreatic-necros* OR WOPN OR walled-off-necros* OR WON OR necrotic-collection* OR cystic-collection*<br>OR<br>pancreatic pseudocyst [Mesh] OR Pancreatic Pseudocysts OR Pseudocyst, Pancreatic OR Pseudocysts, Pancreatic

	AND
	Lumen-apposing-metal-stent OR lumen-apposing metallic-stent OR LAMS OR AXIOS

Table S2 Adverse events rate of Lumen-Apposing Metal Stents (LAMS)

Complications	N (%)
Infection/Occlusion	66 (7.2%)
Bleeding	47 (5.1%)
Migration	23 (2.5%)
Perforation	10 (1.1%)
Stent dislodgement/displacement/misplacement	10 (1.1%)
Abdominal pain	5 (0.5%)
Unplanned endoscopy	5 (0.5%)
Additional percutaneous drain	4 (0.4%)
Stricture	3 (0.3%)
Buried stent	2 (0.2%)
Partially embedded stent	2 (0.2%)
Pneumoperitoneum	2 (0.2%)
Others	6 (0.7%)
Total	185(20.0%)

Table S3 Major adverse events of the meta-analysis

	Bleeding		Infection/Occlusion		Migration	
Author, year, country	LAMS	Plastic stent	LAMS	Plastic stent	LAMS	Plastic stent
Lang GD et al.[14], 2017, USA	4/19	1/84	-	-	-	-
Siddiqui AA et al.[10], 2016, USA	6/86	2/106	4/86	28/106	0/86	3/106
Brimhall B et al.[13], 2018, USA	15/97	5/152	2/97	6/152	-	-
Bang JY et al.[26], 2016, USA	-	-	2/20	5/40	2/20	1/40
Bang JY et al.[12], 2018, USA	4/31	0/29	-	-	1/31	2/29
Total	29/233	8/371	8/203	39/298	3/137	6/175

LAMS, Lumen-Apposing Metal Stents
